# Control of Corrosion Resistance and Osteoclastic Resorbability of Bioresorbable Carbonate Apatite Coating for Biodegradable Mg Alloys through Carbonate Content

**DOI:** 10.1021/acsbiomaterials.5c01706

**Published:** 2025-12-01

**Authors:** Sachiko Hiromoto, Kazuma Midorikawa, Tomohiko Yamazaki, Tomoyuki Yamamoto

**Affiliations:** † Research Center for Structural Materials, 52747 National Institute for Materials Science, Tsukuba 305-0047, Japan; ‡ Faculty of Science and Engineering, 73680 Waseda University, Tokyo 169-8555, Japan; § Research Center for Macromolecules and Biomaterials, National Institute for Materials Science, Tsukuba 305-0047, Japan; ∥ Kagami Memorial Research Institute for Materials Science and Technology, Tokyo 169-0051, Japan

**Keywords:** carbonate apatite coating, carbonate content, corrosion, bioresorption, bioabsorbable/biomedical magnesium alloys, calcium phosphate

## Abstract

To investigate the effect of carbonate content on the corrosion resistance and osteoclastic resorbability of carbonate apatite (CAp) coatings for biodegradable Mg alloys, polarization, electrochemical impedance, and osteoclast precursor cell culture tests were conducted for CAp-coated pure Mg (Mg) and Mg–4Y-3RE (WE43) containing approximately 11, 17, and 18 wt % carbonate. In Hanks’ solution, the polarization resistance (*R*_p_) was higher than in a 0.9% NaCl solution, and the CAp coatings improved the *R*_p_ of Mg by 7 to 15 times. The *R*_p_ of CAp-coated Mg increased by approximately 1.5 times in a 0.9% NaCl solution and 2 times in Hanks’ solution with increasing carbonate content, indicating a reduction in coating defects. For CAp-coated Mg, osteoclasts only survived on the higher carbonate content coating. For WE43, the coating with a higher carbonate content exhibited a higher number of mature osteoclasts and approximately a 1.5-fold increase in the resorbed area by osteoclasts. These findings demonstrate that the carbonate content in the CAp coating allows for adjustment of the corrosion rate of biodegradable Mg alloys to suit the affected part of the body. It was also found that once osteoclasts are induced, the CAp coating with a higher carbonate content is resorbed more quickly by the osteoclasts.

## Graphical abstract

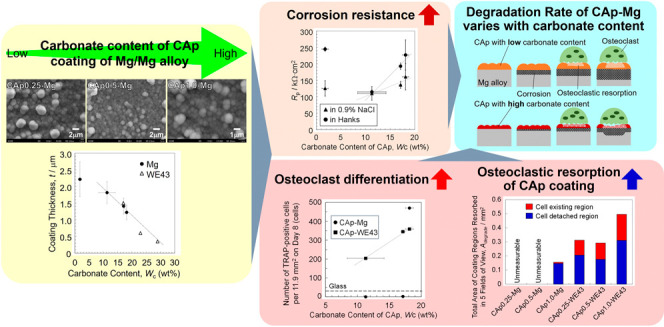

## 1 Introduction

Magnesium (Mg) and Mg alloys are a highly potential biodegradable metallic material as well as Zn and Fe alloys for orthopedic, dental, and circulatory devices, such as bone fixation screws and plates, pins, staples, mesh plates, stents, and so on, due to their good biocompatibility and mechanical compatibility.^1–9^ However, Mg/Mg alloys sometimes show too rapid corrosion in the early stages of implantation, leading to early degradation of mechanical integrity, gas cavity formation due to hydrogen (H_2_) gas, and pH increase caused by OH^–^ ions.^10–17^ Gas cavities may interfere with tissue generation, although the cavities eventually disappear as they are filled with the surrounding tissues.^18^ The healing time of an affected body part depends on its location, as well as on the patient’s health condition and age, since bone healing can be delayed by factors such as aging, a smoking history, and underlying conditions such as diabetes.^19,20^ Mg alloy devices are expected to be used in various anatomical sites, including around bone, inside blood vessels, and in the intestines, each of which presents a distinct corrosion environment. The required periods for medical devices to provide load-bearing or suture retention depend on factors such as anatomical site, patient condition, and local corrosion environment. Therefore, the corrosion rate of the Mg alloy devices should be adjusted accordingly.

To suppress the corrosion of Mg/Mg alloys, various types of coatings, such as calcium phosphate, other phosphate compounds, MgF_2_, bioglasses, TiO_2_, and composite coatings, have been developed. These coatings are typically fabricated using methods such as chemical conversion, microarc oxidation (MAO), sol–gel processing, and ion implantation.^3,6,8,21–29^ In particular, calcium phosphate coatings, such as hydroxyapatite (HAp), β-tricalcium phosphate (β-TCP), calcium phosphate dihydrate (DCPD), and amorphous calcium phosphate coatings, have received significant attention owing to their excellent biocompatibility. Among calcium phosphates, HAp and carbonate apatite (CAp) have drawn special interest for the following reasons. HAp is used as an artificial bone substitute and as a bone conductive coating for artificial joints and dental implants.^30–32^ Additionally, CAp was recently approved as a bioresorbable artificial bone substitute as Cytrans Granules (GC Corporation, Tokyo, Japan),^33^ and a Ca-deficient CAp coating is used for Ti alloy devices.^34,35^ Furthermore, CAp can be resorbed by osteoclasts in hard tissue and replaced with bone^33^ and can also be resorbed by osteoclast-like cells in soft tissue.^36^ Therefore, the CAp coating is expected to function as a bioresorbable coating that provides effective corrosion protection for substrate Mg alloys until osteoclasts or osteoclast-like cells are induced and secrete acids to resorb CAp.

We have developed HAp and CAp coatings whose crystallinity, thickness, and carbonate content can be varied by coating conditions such as the pH, temperature, and carbonate salt concentration of the coating solution and coating time^37–39^ and revealed that the corrosion resistance of the HAp-coated Mg alloys varied with the coating conditions.^40–42^ In a rabbit femoral implantation study for CAp-coated Mg–Ca alloy plates and screws, we found that dissolution of the CAp coating occurred at the surfaces where new bone had formed.^43^

In the CAp coatings, the thickness of the CAp layer decreases with increasing its carbonate content,^38,39^ and it has been reported that the solubility of CAp increases as the carbonate content rises.^44,45^ Therefore, adjusting the carbonate content is expected to offer a means of controlling the corrosion of CAp-coated Mg alloys. To achieve effective regulation via carbonate content, it is crucial to understand the effect of the carbonate content on the corrosion resistance and the osteoclastic resorption behavior of the CAp coating on the surfaces of Mg and its alloys. The resorption behavior of the CAp coating may be influenced by the corrosion of the underlying Mg substrate through an increase in pH since Mg corrosion raises the surface pH, while osteoclastic resorption of CAp is facilitated by the acid produced by osteoclasts. Previously, we reported that the CAp coating with the higher carbonate contents can possibly show higher corrosion resistance for Mg–4Y-3RE (WE43) in the medium,^46^ while the effect of the carbonate content on the osteoclastic resorption behavior was not clarified. It was found that, with carbonate ion source concentrations of 0.4, 1.1, and 1.9 mol/L in this previous study,^46^ the carbonate content of the CAp coating was saturated at 20–25 wt % at concentrations above 1.0 mol/L.^39^ Moreover, this carbonate content range is higher than the 10 to 20 wt % found in bone apatite.^47^ Therefore, it should be said that the previous concentrations were not necessarily suitable for investigating the influence of the carbonate content.

In this study, the effect of the carbonate content in the CAp coating on the corrosion behavior and osteoclastic resorption ability was investigated using CAp-coated pure Mg samples with approximately 11, 17, and 18 wt % carbonate contents. Polarization, electrochemical impedance (EI), and osteoclast cell culture tests were performed. An HAp coating was used as a comparison in the electrochemical corrosion tests. In osteoclast cell culture tests, CAp-coated WE43 samples were additionally used because the corrosion rate of CAp-coated pure Mg was sometimes too high to allow cell evaluation. The composition and structure of the CAp coatings used in this study have already been investigated in detail in our previous publication^39^ and thus are not repeated here.

## 2 Materials and Methods

### 2.1 CAp and HAp Coating of Pure Mg and WE43

Pure Mg (99.95%, Osaka Fuji Corp.) and Mg–4Y-3RE (WE43) (Magnesium Electron) disks with a diameter of 15 or 16 mm and a thickness of 2 mm were prepared from extruded rods. The surface of disks was ground with SiC papers (PSI, USA) up to #1200 and rinsed ultrasonically in 2-propanol. The compositions of pure Mg and WE43 are shown in Table 1.

**Table 1:** Chemical Composition of Pure Mg and WE43 (mass %)

material	Y	Nd	RE^a^	Fe	Si	Ni	Cu	Al
pure Mg				0.0023	0.005	0.0002	0.0002	0.0049
WE43	4.0	2.3	1.1	0.001		0.000	0.002	
								
	Cl	Pb	Zn	Zn + Ag	Li	Zr	IP^b^	Mg
pure Mg	0.002	0.001	0.0055					Bal
WE43				0.03	0.1	0.48	<0.01	Bal

a RE: rare earths.

b IP: impurities.

The CAp coatings were prepared according to the procedure described in our previous study.^39^ A solution containing 0.2 mol/L ethylenediaminetetraacetic acid calcium disodium salt hydrate (C_10_H_12_CaN_2_Na_2_O_8_·*x*H_2_O) solution, 0.2 mol/L potassium dihydrogen phosphate (KH_2_PO_4_), and 0.2 mol/L NaOH was prepared, and the pH was checked to be between 8 and 9. Then, sodium hydrogen carbonate (NaHCO_3_) was added to this solution to obtain final concentrations of 0, 0.25, 0.5, and 1.0 mol/L. Pure Mg and WE43 disks were immersed in the coating solutions at 363 K for 3.6 ks. The coated samples were generally named according to the NaHCO_3_ concentrations and the substrate alloys as HAp-Mg, CAp0.25-Mg, CAp0.5-Mg, and CAp1.0-Mg and CAp0.25-WE43, CAp0.5-WE43, and CAp1.0-WE43. The carbonate content of CAp0.25, CAp0.5, and CAp1.0 coatings was approximately 11, 17, and 18 wt %, respectively, for pure Mg and WE43, according to the previous study, since it was found that the carbonate content is independent of the substrate type.^39^

Surface and cross sections of the coated samples were observed using scanning electron microscopy (SEM) (JSM6500F, JEOL). The crystal structure of the coatings was identified with X-ray diffraction (XRD) (Smart-Lab, RIGAKU) using Cu Kα radiation (λ = 0.15406 nm).

### 2.2 Potentiodynamic Polarization and EI Tests of HAp- and CAp-Mg

Potentiodynamic polarization and EI tests were carried out for HAp- and CAp-Mg in 0.9% NaCl and Hanks’ solutions at 310 K. Each specimen disk was mounted on a specimen holder, exposing 1 cm^2^ to the electrolyte. Ag/AgCl (3 M KCl) and Pt electrodes were used as the reference and counter electrodes, respectively. In the anodic and cathodic polarization tests, open circuit potential (OCP) of the specimen was measured for 1.8 ks until the potential became nearly constant; then, the potential was swept from 50 mV more negative or more positive than the OCP in the anodic or cathodic direction, respectively, at a potential sweep rate of 1 mV s^–1^. In the EI tests, after the 1.8 ks-OCP measurement, the potential perturbation was applied with an amplitude of 5 mV at frequencies from 10 MHz to 10 mHz and with points per decade of 5. An equivalent electric circuit was assumed, and curve-fitting for the measured EI spectra was performed to obtain the values of parameters in the circuit using Zview (Scriber). Then, the polarization resistance (*R*_p_) was calculated from the parameters in the circuit.

### 2.3 Osteoclast Cell Culture on CAp-Mg and WE43

Rat osteoclast precursors (OSC11, Cosmo Bio, Tokyo, Japan) were cultured on CAp-Mg and WE43 with different carbonate contents. Glass disks with a diameter of 13 mm (Matsunami glass, Osaka, Japan) were used as the reference sample. The specimens were dipped in acetone for 10 s for sterilization and dried in air. On Day 0, before seeding cells, each specimen was placed in the well of a 12-well plate and immersed in 2 mL of α-minimum essential medium (α-MEM; Thermo Fisher Scientific, Waltham, Massachusetts, USA) supplemented with 10% fetal bovine serum (FBS; Sigma-Aldrich, St Louis, Missouri, USA) at 310 K for 24 h in a 5% CO_2_ incubator. On Day 1, the used medium was collected from each well, and osteoclast precursor cells were seeded at 4 × 10^4^ cells/well with 4 mL of osteoclast culture medium (OSCMR, Cosmo Bio) containing 50 ng/mL macrophage colony stimulating factor and 15 ng/mL receptor activator of the NF-κB ligand (RANKL). RANKL induces the differentiation of osteoclast precursor cells to mature osteoclast cells. As osteoclast precursors differentiate and mature into osteoclasts, they become multinucleated and increase in size. Once matured, osteoclasts form an actin ring on the bone surface to which they adhered, producing acids within the ring to dissolve the bone. The cells were cultured for 3, 7, and 14 days (up to Days 4, 8, and 15) with a refreshment of osteoclast culture medium on Day 8.

On Days 4, 8, and 15, the cells were dehydrated and fixed to the specimen surface with methanol, and then the cell bodies were stained with Giemsa (Muto Pure Chemicals, Japan) to observe the cell proliferation on the entire surface of the specimen disks. Concurrently, tartrate-resistant acid phosphate (TRAP) in differentiated osteoclast cells was stained using a TRAP staining kit (AK04F, Cosmo Bio) to confirm the differentiation of precursors to osteoclasts. The Giemsa-stained and TRAP-stained specimens were observed using an optical microscope (VHX-5000, Keyence, Japan). The surface morphology of the TRAP-stained specimens was analyzed using a one-shot 3D profilometer (KEYENCE VR-3100). On Day 15, the cytoplasm of living cells and the nuclei of dead cells were stained with calcein in green and propidium iodide in red, respectively. The calcein-PI-stained cells were observed by using a fluorescence microscope (DMIL-TR/EC3, Leica). Magnesium ions in the used medium collected on Days 1, 4, 8, and 15 were quantified by a colorimetric method using Magnesium B-test Wako (Wako Pure Chemical, Osaka, Japan). Three specimens from each sample were used for each cell culture period, and one specimen from each sample was used for Giemsa and TRAP staining, respectively. More than three specimens from each condition were used for quantification of Mg^2+^ ions.

The number of TRAP-positive cells and the mean cell size were obtained from optical images as follows. The optical images of 1.81 × 1.31 mm (2.46 mm^2^) were taken at five fields of view at a magnification sufficient to distinguish cells from corrosion products. Then, the total number of TRAP-positive cells and the total area occupied by the cells in each field of view were determined using ImageJ (NIH). Subsequently, the values for each of the five fields of view were summed. The area occupied by cells was then divided by the number of cells to obtain the mean cell area. In addition, the area of the CAp coating resorbed by osteoclasts was measured using 3D images of the TRAP-stained specimens and further analyzed using low-magnification 3D images of the Giemsa-stained specimens. The measurement procedure is described later.

## 3 Results and Discussion

### 3.1 CAp Coating of Mg and WE43

Figure 1a–g shows the surface and cross-sectional SEM images of HAp- and CAp-Mg and the surface SEM images of CAp-WE43. Figure 1h shows the thicknesses of the HAp and CAp coatings obtained from the cross-sectional SEM images as a function of the carbonate content. Both on Mg and WE43, the CAp coating was composed of one layer of densely agglomerated dome-shaped particles consisting of submicrometer-sized particles. The thickness of the CAp coating decreased from approximately 1.8 to 1.1 μm with an increase in the carbonate content. The HAp coating composed of two layers of a dense inner layer and a porous outer layer with rod-shape particles, and the thickness was about 2.2 μm. These results are consistent with the previous work.^38,39^

**Figure 1:**
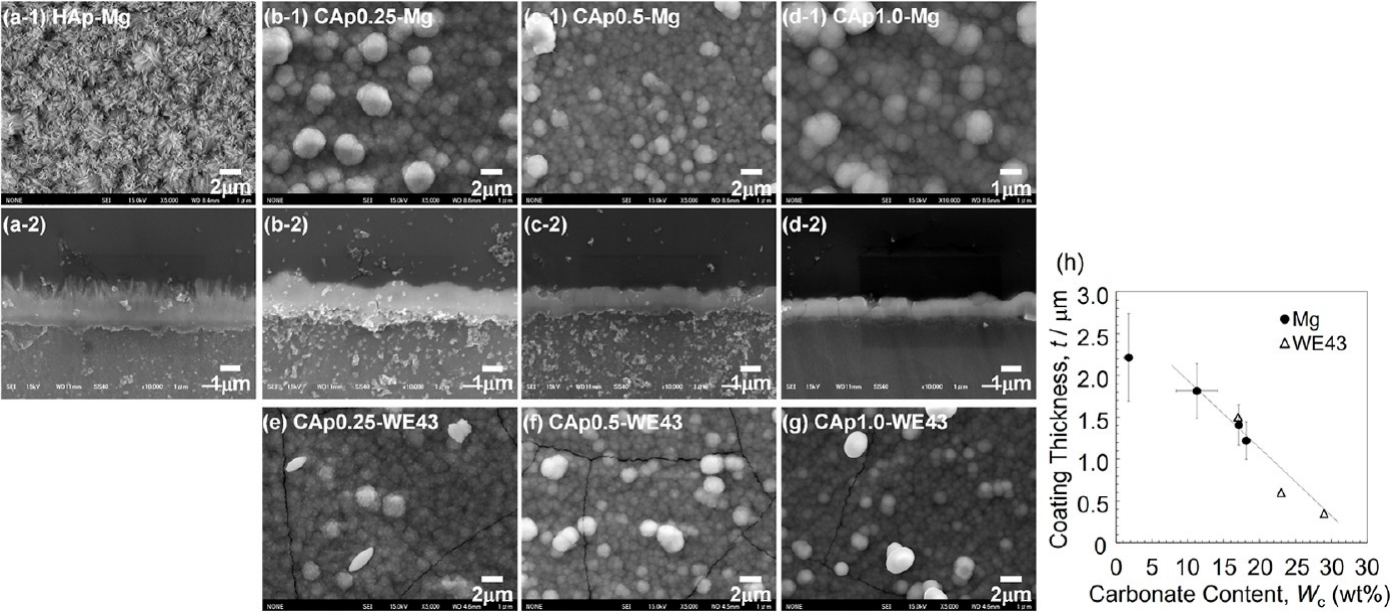
(a-1)–(d-1) and (e–g) Surface and (a-2)–(d-2) cross-sectional SEM images of (a) HAp-Mg, (b) CAp0.25 Mg, (c) CAp0.5 Mg, (d) CAp1.0 Mg, (e) CAp0.25-WE43, (f) CAp0.5-WE43, and (g) CAp1.0-WE43. (h) Coating thickness as a function of the carbonate content of HAp- and CAp-Mg. The coating thickness of CAp-WE43 is cited from ref 38. Adopted from ref 39, Figure 1, with permission.

Figure 2a-1 and b-1 shows the wide-range XRD patterns, and Figure 2a-2 and b-2 shows the magnified apatite 002 plane peak. For all the samples, diffraction peaks from the apatite structure were observed at around 26, 28, 29, 33, 49, 51.5, and 53°. CAp-Mg exhibited a shift in the apatite 002 plane peak position at each NaHCO_3_ concentration similar to that observed for CAp-WE43. This result indicates that the CAp coating on Mg had the same carbonate content as that on WE43. The presence of carbonate groups within the apatite structure was previously confirmed by Fourier transform infrared absorption measurements,^39^ as shown in Figure S1.

**Figure 2:**
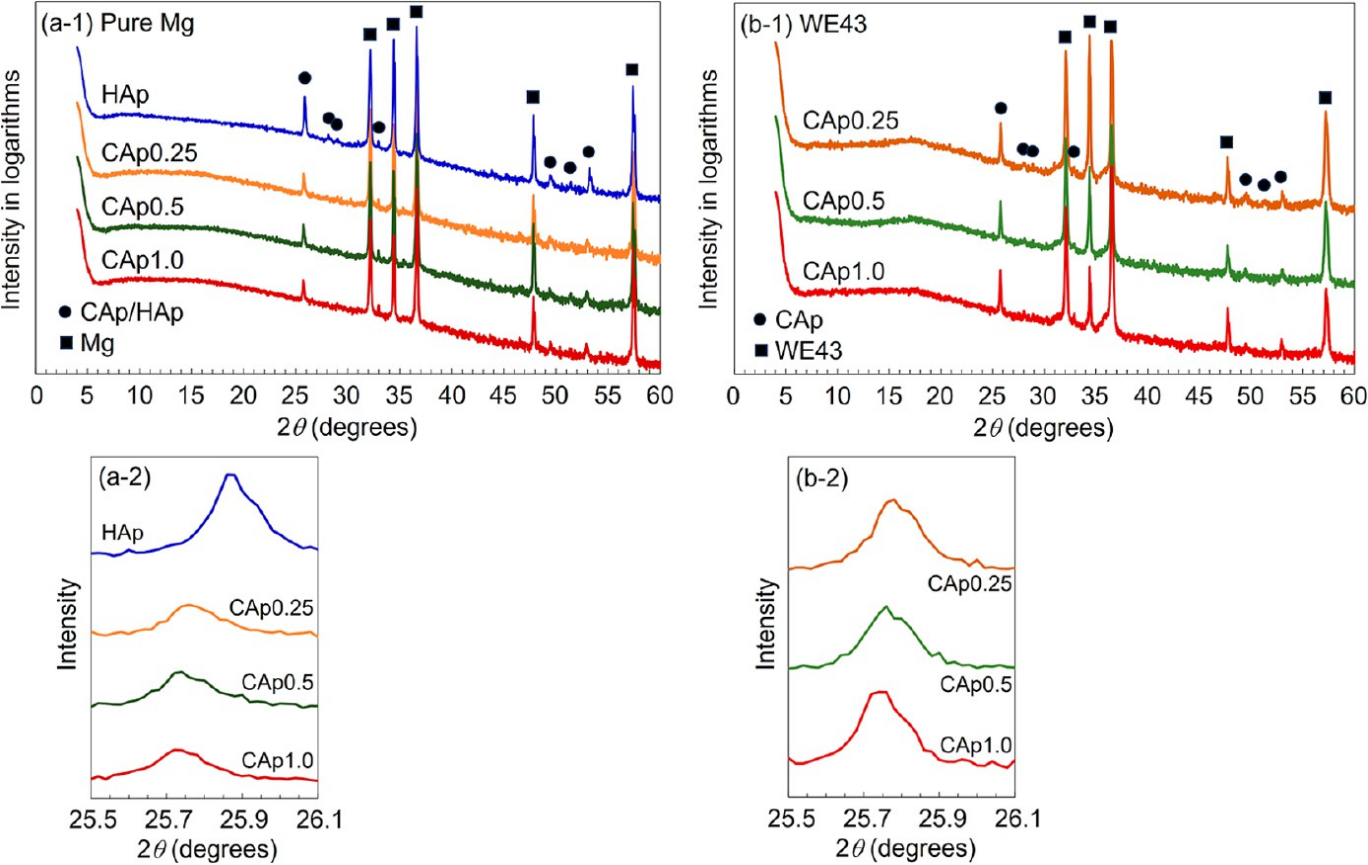
XRD patterns and magnified apatite 002 plane peak of (a) HAp- and CAp-Mg and (b) CAp-WE43. (a-1) and (b-1) Wide-range XRD pattern and (a-2) and (b-2) magnified apatite 002 plane peak.

### 3.2 Polarization Behavior of HAp- and CAp-Mg

Figure 3a–d shows the anodic and cathodic polarization curves of HAp- and CAp-Mg in 0.9% NaCl and Hanks’ solutions. Corrosion current density (*I*_corr_) obtained from the polarization curves is shown in Figure 3e. In a 0.9% NaCl solution, the cathodic current density of CAp-Mg was higher than that of HAp-Mg, and it increased with an increase in the carbonate content (Figure 3a). The anodic current density near the corrosion potential (*E*_corr_) of CAp-Mg was apparently higher than that of HAp-Mg. Only CAp0.25 Mg exhibited a constant current region, followed by a rapid current increase due to the breakdown of the CAp coating, whereas the other samples showed a gradual current increase from the *E*_corr_ up to the breakdown potential.

**Figure 3:**
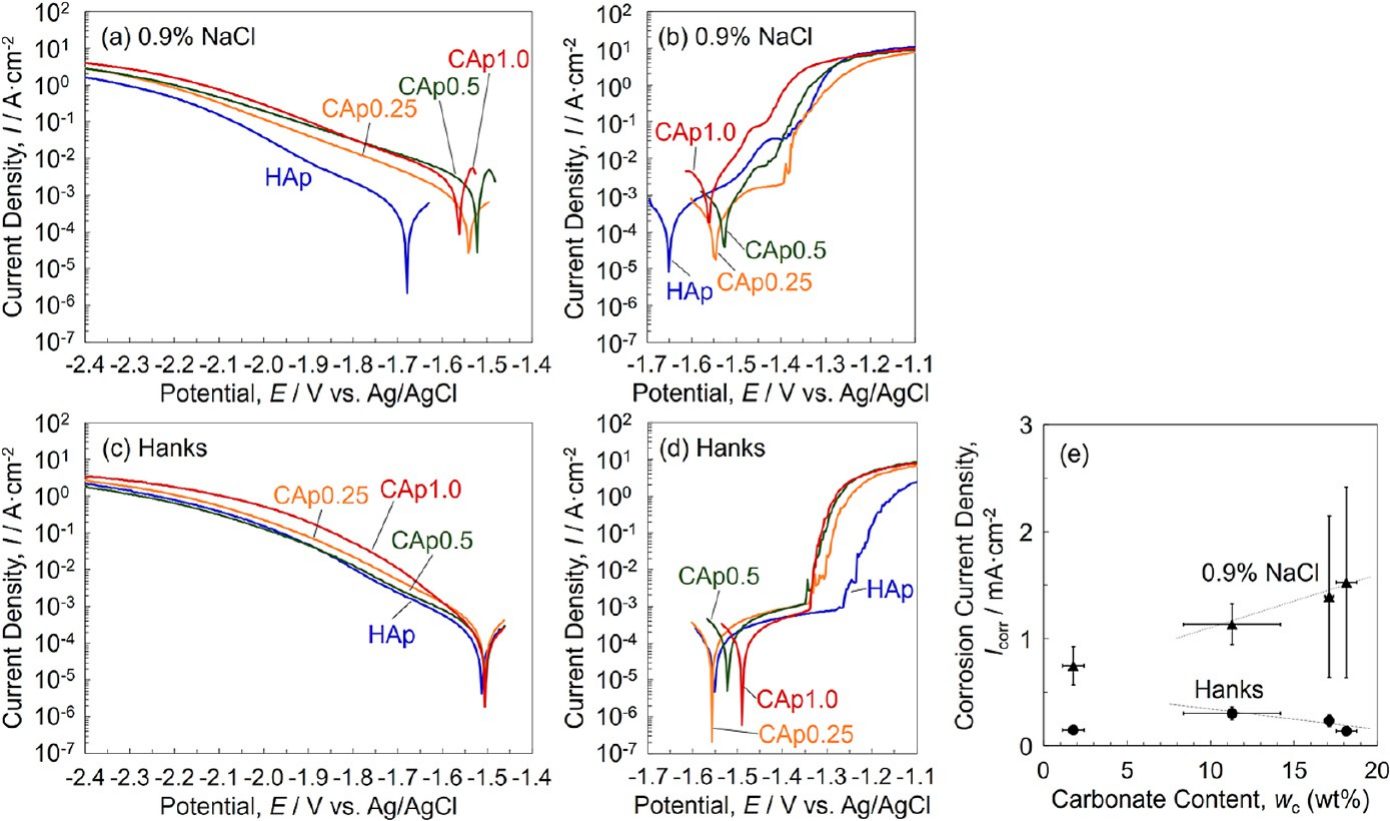
Potentiodynamic (a) and (c) cathodic and (b) and (d) anodic polarization curves of HAp- and CAp-Mg in (a) and (b) 0.9% NaCl and (c) and (d) Hanks’ solutions. (e) *I*_corr_ as a function of the carbonate content of CAp-Mg.

In Hanks’ solution, the cathodic current density near the *E*_corr_ decreased in comparison to that in a 0.9% NaCl solution for CAp-Mg. The anodic polarization curves in Hanks’ solution showed a clear constant current region with a lower current density value than in a 0.9% NaCl solution, leading to the lower *I*_corr_ values in Hanks’ solution than those in a 0.9% NaCl solution (Figure 3e). After anodic polarization, several large corrosion pits were formed on the specimen surfaces in both solutions. The SEM observation and energy-dispersive X-ray spectroscopy (EDS) analysis revealed that in a 0.9% NaCl solution, cracks and delamination of the HAp and CAp coatings were observed over almost the entire surface, and the surface was covered with Mg­(OH)_2_ corrosion products (Figure S2a,c and Table S1). In contrast, in Hanks’ solution, cracks and delamination of the coatings were not observed outside the corrosion pits, and corrosion products containing calcium phosphate were formed in the corrosion pits (Figure S2b,d and Table S1). It was reported that calcium phosphates can readily precipitate as Mg corrodes in Hanks’ solution.^8,48^ These facts indicate that calcium phosphate deposited to fill the defects in the HAp and CAp coatings in Hanks’ solution, leading to the clear constant current region (Figure 3d).

With an increase in the carbonate content, the *I*_corr_ in 0.9% NaCl increased, whereas that in Hanks’ solution slightly decreased (Figure 3e). The higher corrosion protectiveness associated with the higher carbonate content in the CAp coating was also suggested for WE43 in medium.^46^ Considering that the CAp coating with the higher carbonate content was thinner (Figure 1) and showed the higher *I*_corr_ in 0.9% NaCl solution (Figure 3e), CAp-Mg with the higher carbonate content presumably exhibited a higher corrosion rate immediately after immersion in Hanks’ solution, promoting the precipitation of calcium phosphates that could repair defects in the thin coating, ultimately enhancing the protective nature.

### 3.3 EI Behavior of HAp- and CAp-Mg

Figure 4a,b shows the Nyquist plots of the EI spectra of HAp- and CAp-Mg in 0.9% NaCl and Hanks’ solutions. In both solutions, HAp- and CAp-Mg showed two flattened semicircles at medium and low frequencies, while the uncoated Mg showed one semicircle. Then, the *R*_p_ of the uncoated Mg in 0.9% NaCl and Hanks’ solutions was determined from the diameter of the semicircle as approximately 25 Ω·cm^2^ and 15 kΩ·cm^2^, respectively.

**Figure 4:**
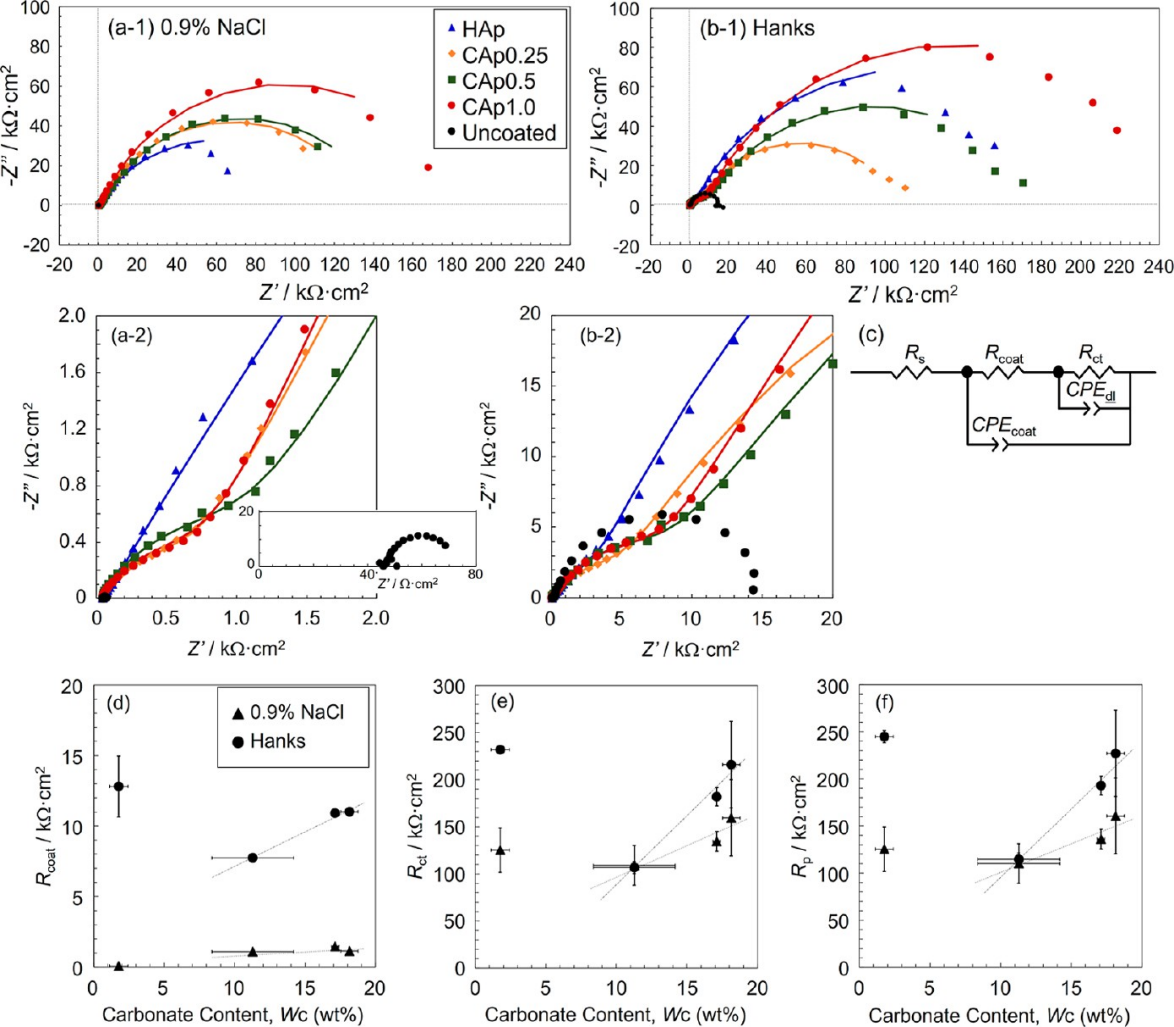
(a) and (b) Nyquist plots of HAp- and CAp-Mg in (a) 0.9% NaCl and (b) Hanks’ solutions and (c) assumed equivalent circuit. (a-2) and (b-2) magnified plots of (a-1) and (b-1). The inset in (a-2) shows the Nyquist plot of uncoated Mg. (d) *R*_coat_, (e) *R*_ct_, and (f) *R*_p_ as a function of the carbonate content of HAp- and CAp-Mg in 0.9% NaCl and Hanks’ solutions obtained by curve-fitting.

An equivalent electric circuit shown in Figure 4c was then assumed for HAp- and CAp-Mg. Constant phase element (*CPE*) was used because of the flattened semicircles on the Nyquist plots and the microscopically rough surface of the apatite coatings (Figure 1). *R*_s_ represents the solution resistance. *R*_coat_ corresponds to the resistance of the apatite coatings (solution resistance inside the apatite coatings), and *CPE*_coat_ represents the *CPE* of the apatite coatings, which depends on the porosity, thickness, and so on. *R*_ct_ and *CPE*_dl_ represent the charge transfer resistance and the *CPE* of the electric double layer, respectively. The *R*_ct_ generally depends on the number and size of defects at the boundary between the coating and substrate. The *R*_p_ is calculated from *R*_coat_ and *R*_ct_, using eq 1.

1 $$\frac{1}{R_{p}}=\frac{1}{R_{coat}+R_{ct}}$$

The circuit parameters were determined through curve fitting, and the fitted curves are shown in Figure 4a,b. The obtained parameters are summarized in Table 2, and *R*_coat_, *R*_ct_, and *R*_p_ are plotted as a function of the carbonate content in Figure 4d–f.

**Table 2:** Parameters in the Assumed Equivalent Electric Circuit

	sample	*R*_s_ (Ω·cm^2^)	*R*_coat_ (kΩ·cm^2^)	*CPE*_coat_-*T* (μF·s^P–1^·cm^2^)	*CPE*_coat_-*P* (−)	*R*_ct_ (kΩ·cm^2^)	*CPE*_dl_-*T* (μF·s^P–1^·cm^2^)	*CPE*_dl_-*P* (−)	*R*_p_ (kΩ·cm^2^)
0.9% NaCl	HAp-Mg	38 ± 3.8	0.81 ± 0.65	4.1 ± 0.002	0.87 ± 0.16	125 ± 23	13 ± 0.16	0.65 ± 0.01	125 ± 24
	CAp0.25 Mg	31 ± 4.6	1.1 ± 0.19	3.0 ± 0.84	0.66 ± 0.02	109 ± 21	6.9 ± 0.072	0.71 ± 0.01	110 ± 21
	CAp0.5 Mg	312 ± 3.0	1.5 ± 0.062	1.4 ± 0.37	0.71 ± 0.01	135 ± 11	7.4 ± 0.58	0.69 ± 0.01	136 ± 10
	CAp1.0 Mg	34 ± 8.2	1.1 ± 0.079	2.1 ± 0.48	0.67 ± 0.02	160 ± 40	4.2 ± 0.34	0.76 ± 0.01	161 ± 40
Hanks	HAp-Mg	31 ± 1.7	13 ± 2.2	11 ± 0.52	0.62 ± 0.00	232 ± 4.4	4.7 ± 0.55	0.87 ± 0.03	245 ± 6.3
	CAp0.25 Mg	27 ± 1.8	7.7 ± 0.31	2.1 ± 0.29	0.67 ± 0.01	107 ± 6.3	8.9 ± 0.38	0.68 ± 0.02	115 ± 6.6
	CAp0.5 Mg	27 ± 5.8	11 ± 0.23	1.0 ± 0.11	0.70 ± 0.01	182 ± 9.6	7.9 ± 0.02	0.65 ± 0.01	193 ± 9.8
	CAp1.0 Mg	26 ± 3.5	11 ± 0.24	1.4 ± 0.32	0.68 ± 0.01	216 ± 46	5.1 ± 0.35	0.75 ± 0.01	227 ± 46

In a 0.9% NaCl solution, the *R*_p_ of Mg was improved more than 5000 times with the CAp coating, from approximately 25 Ω·cm^2^ to 110–160 kΩ·cm^2^. Both *R*_coat_ and *R*_ct_ slightly increased with the carbonate content, resulting in an approximately 1.5-fold increase in *R*_p_. As shown in Table 2, both *CPE*_coat_-*T* and *CPE*_dl_-*T* tended to decrease with increasing carbonate content. The increase in *R*_coat_ and *R*_ct_ and the decrease in *CPE*_coat_-*T* and *CPE*_dl_-*T* indicate a reduction in coating defects. However, the increase in *R*_p_ with the carbonate content was inconsistent to that of the *I*_corr_ (Figure 2e). This discrepancy between *R*_p_ and *I*_corr_ might be influenced by the characteristic known as the anomalous hydrogen evolution of Mg.^49^ The details remain a subject of future investigation.

In Hanks’ solution, the *R*_p_ of Mg was improved by more than 7 times with the CAp coating, from approximately 15 kΩ·cm^2^ to 110–230 kΩ·cm^2^. Both *R*_coat_ and *R*_ct_ increased with an increasing carbonate content, resulting in an approximately 2-fold increase in *R*_p_. Both *CPE*_coat_-*T* and *CPE*_dl_-*T* tended to decrease with an increasing carbonate content (Table 2). The change in these impedance parameters indicates a reduction of coating defects. Eventually, it can be said that the corrosion protectiveness of the CAp coating increased with increasing carbonate content due to the decrease in coating defects.

The *R*_coat_ and *R*_ct_ of CAp-Mg were higher in Hanks’ solution than those in a 0.9% NaCl solution, indicating that the solution permeability and defects in the CAp coating in Hanks’ solution were smaller than those in a 0.9% NaCl solution. SEM-EDS analysis after EI measurements showed the deposition of calcium phosphate on the CAp1.0 coating in Hanks’ solution (Figure S3 and Table S2), suggesting that the defects in the CAp coating were repaired with deposited calcium phosphate. As a result, the *R*_p_ of CAp-Mg in Hanks’ solution became higher than in a 0.9% NaCl solution. This result was consistent with that of *I*_corr_ (Figure 3e).

The lowest *R*_coat_ of HAp-Mg in a 0.9% NaCl solution indicates that the number and size of defects in the HAp coating were larger than those in the CAp coating. In contrast, in Hanks’ solution, HAp-Mg exhibited the *R*_p_ equivalent to that of CAp1.0 Mg. Deposition of corrosion products containing calcium phosphate on HAp-Mg during anodic polarization in Hanks’ solution (Figure S2 and Table S1) suggests that the defects in the HAp coating were repaired by these corrosion products. However, no significant change in the surface morphology or composition of the HAp coating was observed before and after EI measurements (Figure S3 and Table S2), suggesting that the amount of deposited calcium phosphate during EI measurements was relatively small.

Consequently, it was found that the corrosion protectiveness of the CAp coating can be controlled by adjusting the carbonate content. This property is promising as a means to tailor the degradation rate of Mg devices depending on the patient’s age, health condition, and so on. On the other hand, the different results obtained in 0.9% NaCl and Hanks’ solutions indicate that the corrosion protectiveness of the CAp coating is influenced by environmental factors, such as calcium phosphate deposition. In addition, we have reported that the corrosion protectiveness of the HAp coating also depends on the composition of the Mg substrate.^50^ Further investigation is required to precisely control the degradation rate of Mg devices in vivo by adjusting the carbonate content of the CAp coating.

### 3.4 Cell Proliferation Behavior on CAp-Mg and WE43

Figure 5 exhibits the whole surface optical images of CAp-Mg and WE43 and the glass with Giemsa-stained cells on Days 4, 8, and 15. Magnified Giemsa-stained images are shown in Figure S4. Calcein-PI fluorescence-stained images on Day 15 are shown in Figure 6. Giemsa stains cells in purple regardless of their viability, and calcein-PI stains living and dead cells in green and red, respectively. The accumulated amount of Mg^2+^ ions released in the medium at each time point during cell culturing, as well as the total amount of Mg^2+^ ions released over 15 days as a function of the carbonate content in the CAp coating, are shown in Figure 7. For CAp-Mg, only CAp0.5- and CAp1.0 Mg on Day 15 obviously exhibited the presence of cells in the Giemsa-stained images (Figure 5a–c and Figure S4a–c). In the calcein-PI-stained images, CAp0.25-Mg showed a few cells, most of which were dead; CAp0.5-Mg showed a small number of cells, about half of which were alive; and CAp1.0-Mg showed numerous cells, most of which were alive (Figure 6a–c). The Mg^2+^ ion release was significant from the beginning of the cell culture for CAp0.25- and CAp0.5-Mg, which increased with culturing period, whereas that from CAp1.0-Mg was suppressed up to Day 4 and gradually began between Days 4 and 8. The total amount of released Mg^2+^ ions of CAp1.0-Mg was about an order of magnitude smaller than that of CAp0.25- and CAp0.5-Mg (Figure 7c). CAp0.25- and CAp0.5-Mg showed obvious corrosion on the disk edges on Day 4, while CAp1.0-Mg showed no obvious corrosion on Day 4. It was thus clearly demonstrated that the higher carbonate content in the CAp coating suppressed the substrate Mg corrosion, leading to a higher cell viable rate. Since most cells died on CAp0.25- and CAp0.5-Mg (Figure 6a–c), the influence of the carbonate content on the osteoclastic response to the CAp coating could not be examined using CAp-Mg samples.

**Figure 5:**
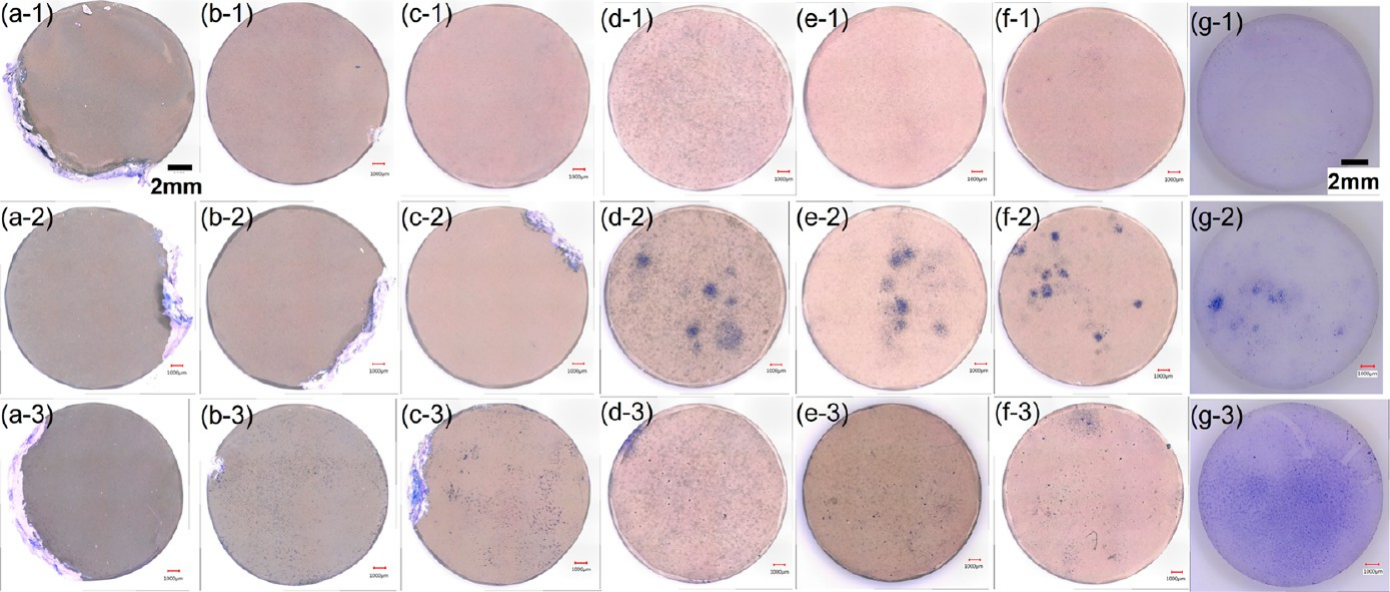
Optical surface images of (a) CAp0.25-Mg, (b) CAp0.5-Mg, (c) CAp1.0-Mg, (d) CAp0.25-WE43, (e) CAp0.5-WE43, (f) CAp1.0-WE43, and (g) glass with Giemsa-stained osteoclast cells. Images on (a-1)–(g-1) Day 4, (a-2)–(g-2) Day 8, and (a-3)–(g-3) Day 15. The scale bar for CAp-Mg and WE43 is shown in image (a-1) and that for glass is shown in image (g-1).

**Figure 6:**
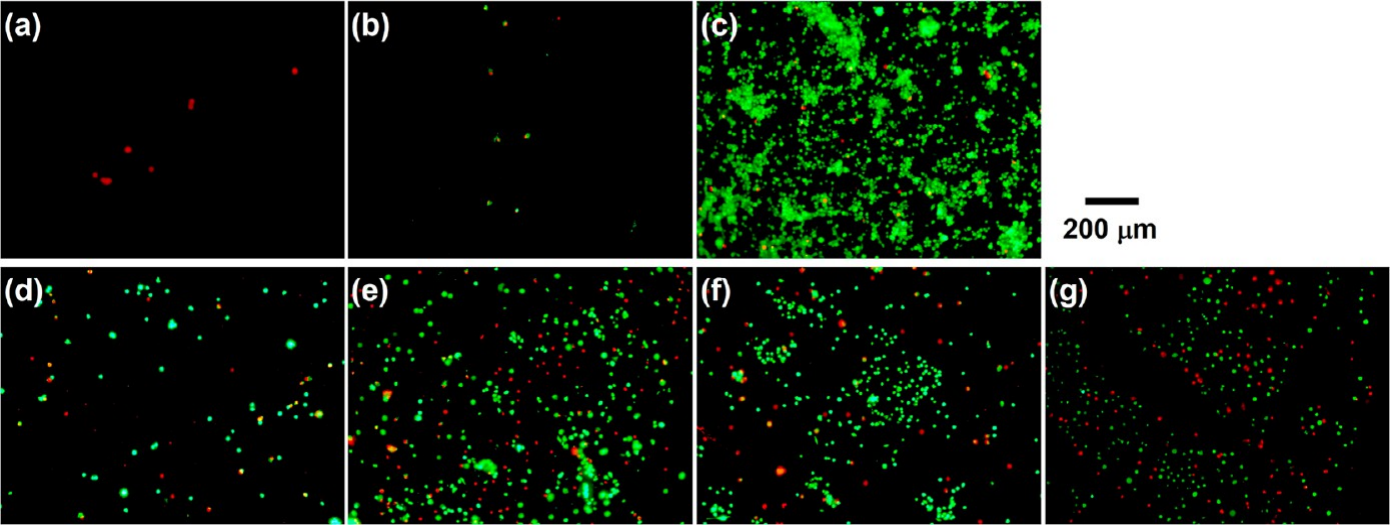
Composite fluorescence images of calcein- and PI-stained osteoclast cells on Day 15 of (a) CAp0.25-Mg, (b) CAp0.5-Mg, (c) CAp1.0-Mg, (d) CAp0.25-WE43, (e) CAp0.5-WE43, (f) CAp1.0-WE43, and (g) glass. Living cells, green; dead cells, red.

**Figure 7:**
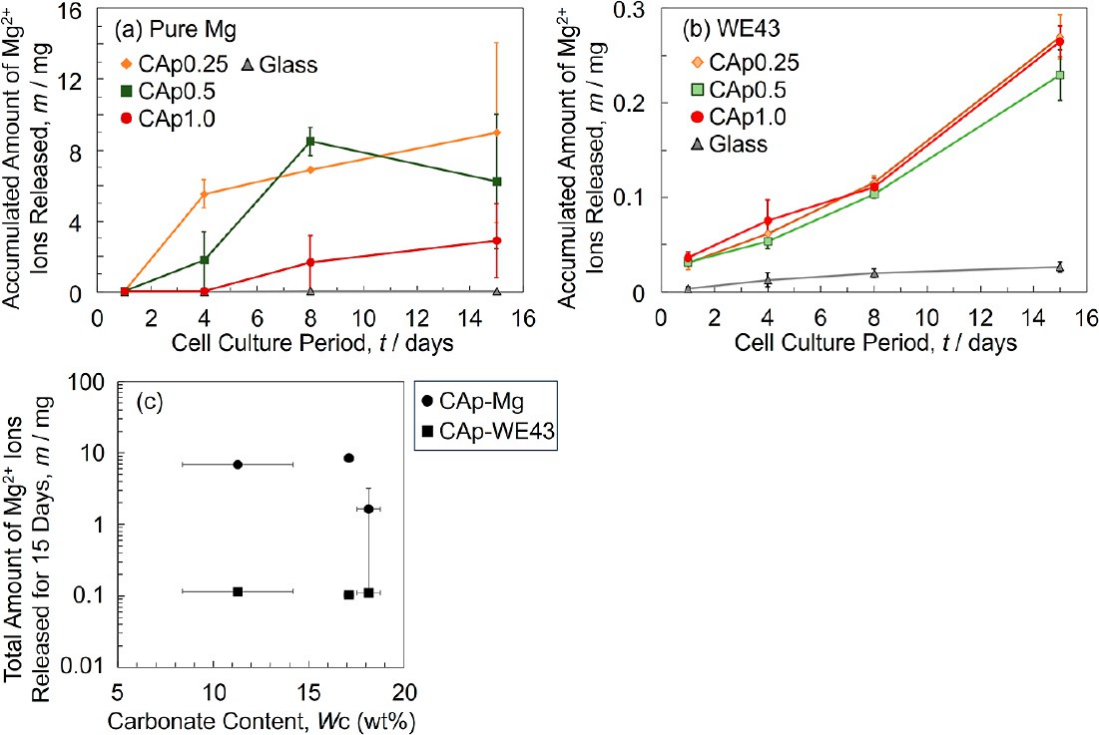
Accumulated amount of Mg^2+^ ions released from (a) CAp-Mg and (b) CAp-WE43 as a function of cell culture period. (c) Total amount of Mg^2+^ ions released for 15 days as a function of carbonate content.

For CAp-WE43 and the glass (Figures 5d–g and S4d–g), the Giemsa-stained images clearly demonstrated cell proliferation and colony formation up to Day 8, followed by the disappearance of colonies by Day 15. In contrast, the cells on the glass appeared to continue increasing up to Day 15 (Figures 5g and S4g). A similar decrease in cell number from Day 8 to 15 was previously observed on the surface of CAp-WE43 with the higher carbonate contents than those in this study.^31^ It was suggested that acids secreted by mature osteoclast cells corroded the underlying WE43 substrate, elevating the surrounding pH and causing the cell detachment.^31^ The calcein-PI-stained images (Figure 6d–g) show that most of the cells on CAp-WE43 were alive to a similar extent to the glass surface. The amount of Mg^2+^ ions released from CAp-WE43 was 1 to 2 orders of magnitude lower than that from CAp-Mg (Figure 7). In addition, no apparent corrosion was observed on CAp-WE43, as shown in Figure 5d–f. Owing to their higher corrosion resistance, cells were able to proliferate on CAp-WE43.

The influence of the carbonate content in the CAp coating on the Mg^2+^ ion release behavior was not significant (Figure 7b,c), although an improvement in the corrosion protectiveness of the CAp coating with increasing carbonate content was shown by the electrochemical tests in 0.9% NaCl and Hanks’ solutions (Figures 3 and 4). Biological corrosion factors, such as acid secretion from osteoclasts, the presence of proteins, and the higher corrosion resistance of WE43 compared to pure Mg, might mask the effect of the carbonate content.

Most cells survived for 15 days, regardless of the carbonate content in the CAp coating of WE43. Therefore, CAp-WE43 was mainly used to investigate the influence of the carbonate content on the osteoclastic response to the CAp coating.

### 3.5 Osteoclastic Response to CAp-WE43

TRAP staining was performed to investigate the differentiation and maturation of osteoclast precursors to osteoclasts. Osteoclast precursors differentiate into mononuclear osteoclasts, which mature into multinucleated giant cells. Differentiated osteoclasts express TRAP that is stained red in TRAP staining. Figure S5 shows the whole surface optical images of CAp-Mg and WE43 and the glass with TRAP-stained cells on Days 4, 8, and 15. The number of TRAP-positive cells was apparently smaller than that of Giemsa-stained cells, and even on CAp-WE43 surfaces at Day 8—where the most distinct colonies were observed with Giemsa staining (Figure 5)—colonies were not clearly observed on the TRAP-stained surfaces (Figure S5). Furthermore, very few TRAP-positive cells were observed on the glass surface. These results indicate that not all precursor cells differentiated and matured into osteoclasts. Typical TRAP-stained images of CAp-WE43 and the glass on Days 4, 8, and 15 are shown in Figure 8a–d. Figure 8e,f presents the total number of TRAP-positive cells in the five fields of view and the mean area per cell (i.e., mean cell size) as a function of the culture period. Figure 9 shows magnified optical images and the corresponding topographic images of relatively large TRAP-positive cells for CAp-Mg and CAp-WE43 on Days 4, 8, and 15.

**Figure 8:**
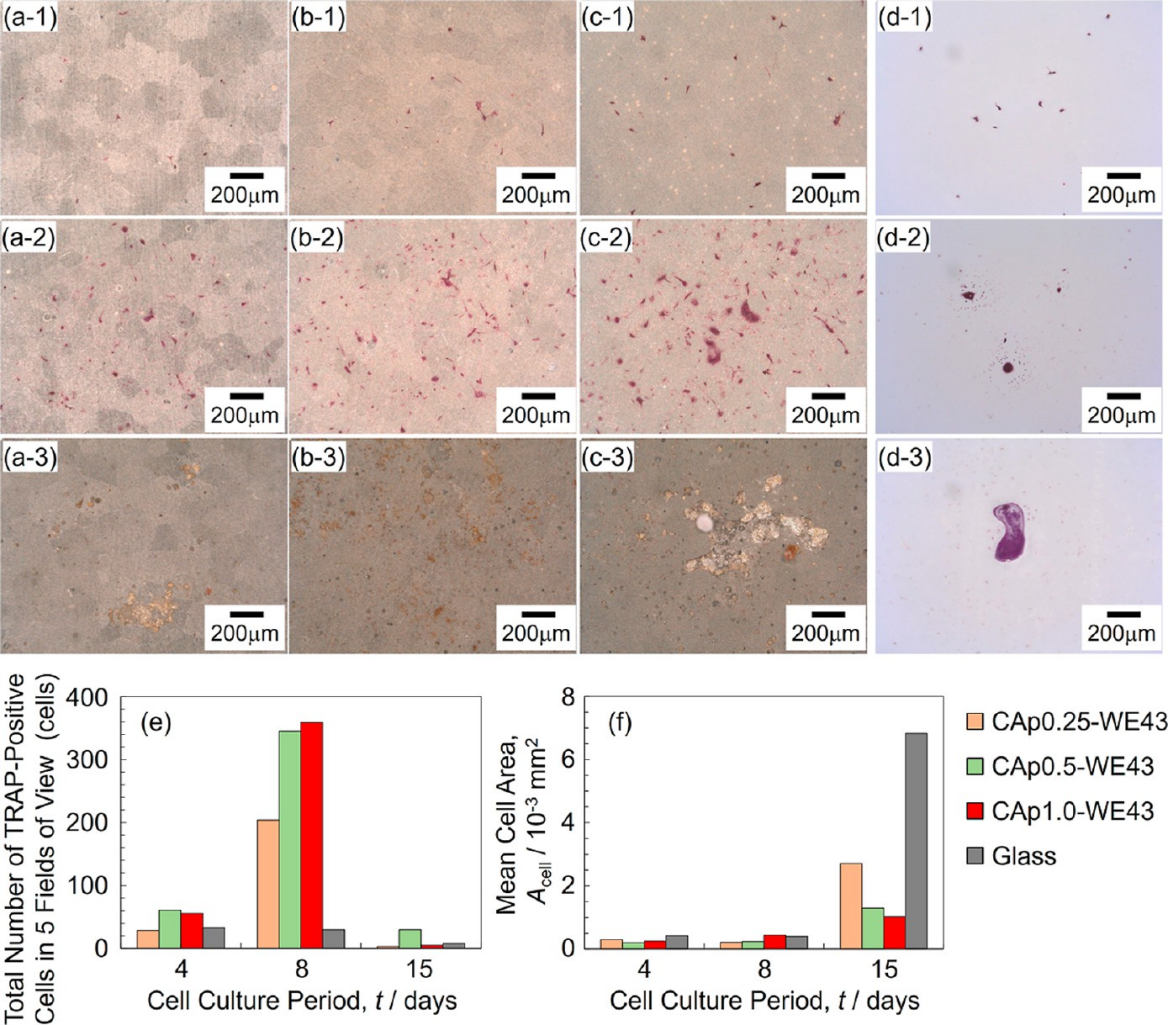
Optical surface images of (a) CAp0.25-WE43, (b) CAp0.5-WE43, (c) CAp1.0-WE43, and (d) glass with TRAP-stained osteoclast cells. Images on (a-1)–(d-1) Day 4, (a-2)–(d-2) Day 8, and (a-3)–(d-3) Day 15. (e) Number of TRAP-positive cells (*n* = 1) and (f) mean cell size as a function of cell culture period (*n* = 1).

**Figure 9:**
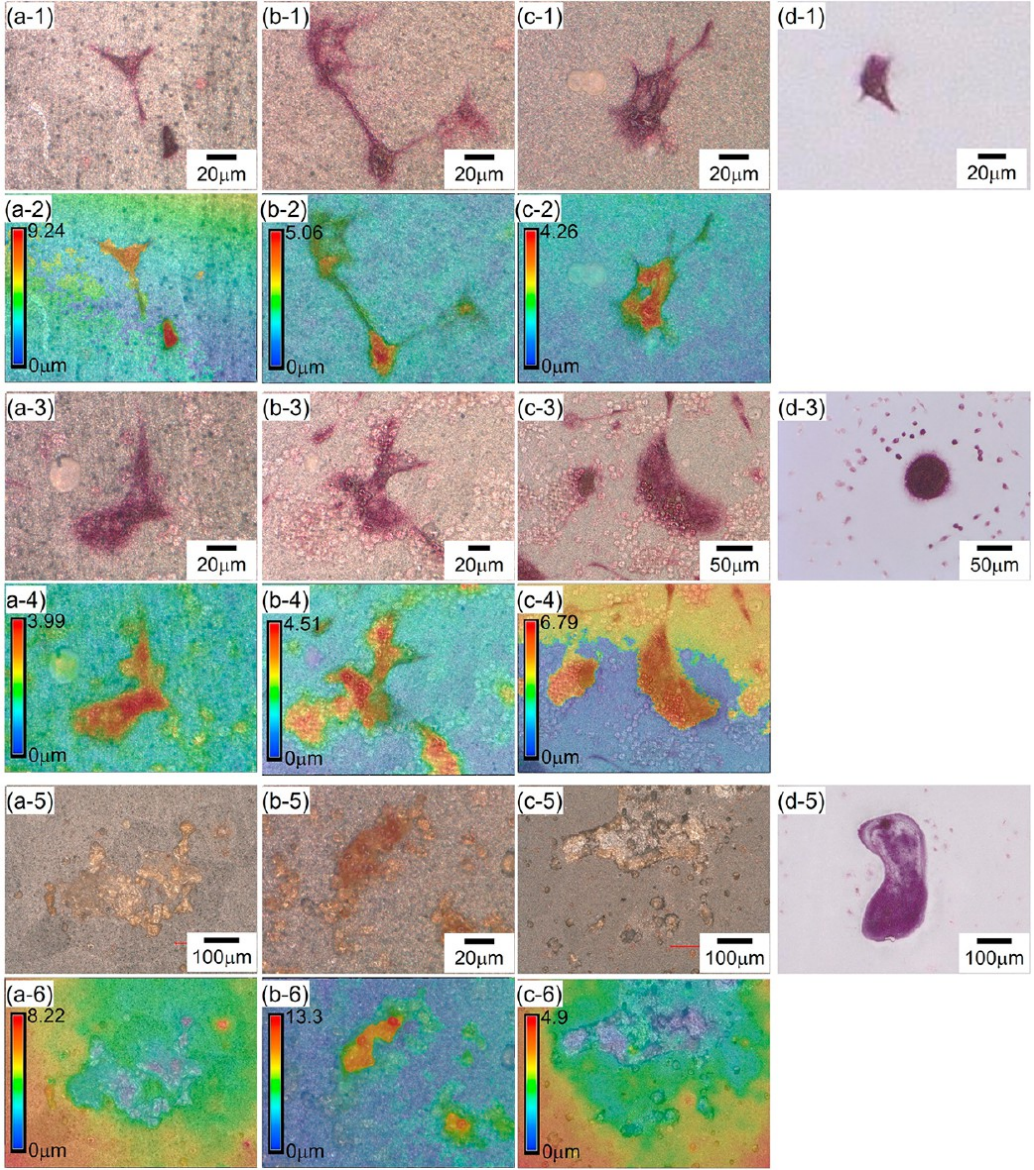
Optical images and corresponding topographic images of (a) CAp0.25-WE43, (b) CAp0.5-WE43, (c) CAp1.0-WE43, and (d) glass with TRAP-stained cells. (a-1)–(d-1), (a-3)–(d-3), and (a-5)–(d-5) Optical images and (a-2)–(d-2), (a-4)–(d-4), and (a-6)–(d-6) topographic images on (a-1)–(d-1) and (a-2)–(d-2) Day 4, (a-3)–(d-3) and (a-4)–(d-4) Day 8, and (a-5)–(d-5) and (a-6)–(d-6) Day 15.

Red-colored TRAP-positive cells were observed from Day 4 for all CAp-WE43 samples, and their number and size were comparable to those on the glass. The number of cells increased significantly up to Day 8 on CAp-WE43 samples but not on the glass (Figure 8e), and this result is consistent with the increase in the cell number observed in the Giemsa-stained CAp-WE43 samples (Figure 5). The mean cell size on CAp-WE43, as obtained from the TRAP-stained images, did not change obviously between Day 4 and Day 8 and was similar to that on the glass (Figure 8f). However, in the Giemsa-stained CAp-WE43 specimens (Figures 5 and S4), two or three giant cells of 200–300 μm were observed in the colonies of CAp0.5- and CAp1.0-WE43 on Day 8, while such giant cells were not found on Day 4. It is considered that due to the large number of small cells present in the TRAP-stained specimens on Day 8, the mean cell size became smaller, even though giant cells were present. On Day 8, TRAP-positive cells were clustered together in colony-like formations on the whole surface images (Figure S5d–f). These results indicate that osteoclast precursors proliferated and differentiated into osteoclasts up to Day 8 on CAp-WE43 samples. On Day 15, the whole surface images (Figure S5) exhibited few relatively large TRAP-positive cells, while faintly red cells were observed in the high-magnification images (Figure 8(a-3)–(c-3) and (e)). On the other hand, the mean cell size increased, and the discolored regions similar in size to the cells appeared in the CAp coating (Figures 8f and 9(a-5)–(c-5)). These facts indicate that the proliferation and multinucleation of osteoclasts progressed up to around Day 8, after which mature osteoclasts each formed an actin ring on the surface to which they adhered and secreted acids within the ring between Days 8 and 15, leading to cell detachment.

The number of TRAP-positive cells on CAp-WE43 samples was clearly greater than that on the glass on Day 8 (Figures 8e and S5). The Giemsa-stained images (Figures 5 and S4) show that CAp0.5- and CAp1.0-WE43 exhibited giant cells within the colonies, whereas such large cells were not observed on the glass. These results indicate that the CAp coating further stimulates osteoclast differentiation in addition to the promotion by RANKL since RANKL in the medium promotes osteoclast differentiation on the glass. On Day 15, mature cells appeared to have detached from the CAp-coated surfaces. Therefore, the larger cell size observed on the glass surface at Day 15 does not imply that the glass surface is more supportive of osteoclast differentiation than the CAp-coated surfaces. Additionally, Fujioka-Kobayashi et al. reported that CAp provides a more favorable surface for osteoclast differentiation compared to deproteinized bovine bone mineral.^51^ These findings suggest that the CAp coating enhances the proliferation and differentiation of osteoclasts, regardless of the carbonate content.

Figure 10 shows the total number of TRAP-positive cells and the mean cell size on Day 8 as functions of the carbonate content in the CAp coating. The results for CAp-Mg are also plotted, although these are based on very small numbers of cells. Both the number and the size of TRAP-positive cells increased with an increasing carbonate content. Additionally, in the Giemsa-stained images, giant cells were observed within the colonies of CAp0.5- and CAp1.0-WE43, whereas no obvious giant cells were observed in CAp0.25-WE43 (Figures 5 and S4). Therefore, these results demonstrate that the proliferation and maturation of osteoclast precursors are promoted by the increased carbonate content of the CAp coating.

**Figure 10:**
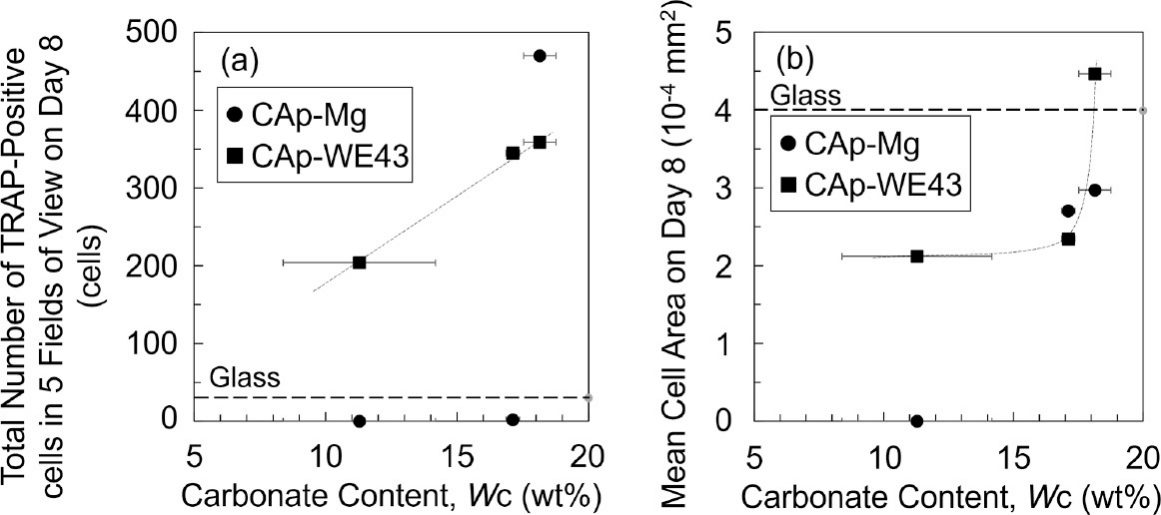
(a) Number of TRAP-positive cells and (b) mean cell size as a function of carbonate content of CAp-Mg and WE43.

### 3.6 Resorption Behavior of the CAp Coating by Osteoclasts

In evaluating the effect of the carbonate content on the osteoclastic resorption of the CAp coating, the following assumptions were made:^1^ All TRAP-positive cells secrete acids and resorb the CAp coating; therefore, osteoclastic resorption occurs beneath the remaining TRAP-positive cells.^2^ According to the topographical images shown in Figure 9, resorption of the CAp coating results in the formation of concave regions on the surface. Thus, surface areas exhibiting concave features with shapes and sizes comparable to those of the cells were regarded as resorbed regions from which the cells had detached. Based on these assumptions, the sum of the areas beneath the remaining TRAP-positive cells and the identified concave regions was taken as the total resorbed area of the CAp coating.

To quantify these areas, 3D images, such as those shown in Figure 11a, were taken for five fields of view in each sample. The areas corresponding to the remaining cells and the concave regions were measured using the analysis software equipped with the one-shot 3D profilometer. These measured areas in the five fields of view were then summed to determine the resorbed area of the CAp coating, as shown in Figure 11b. The resorbed area of the CAp coating on WE43 was approximately 1.5 times larger for CAp1.0 than for CAp0.25 and CAp0.5 coatings. On pure Mg, osteoclastic resorption was clearly observed for the CAp1.0 coating, which allowed the cell proliferation and osteoclastic differentiation.

**Figure 11:**
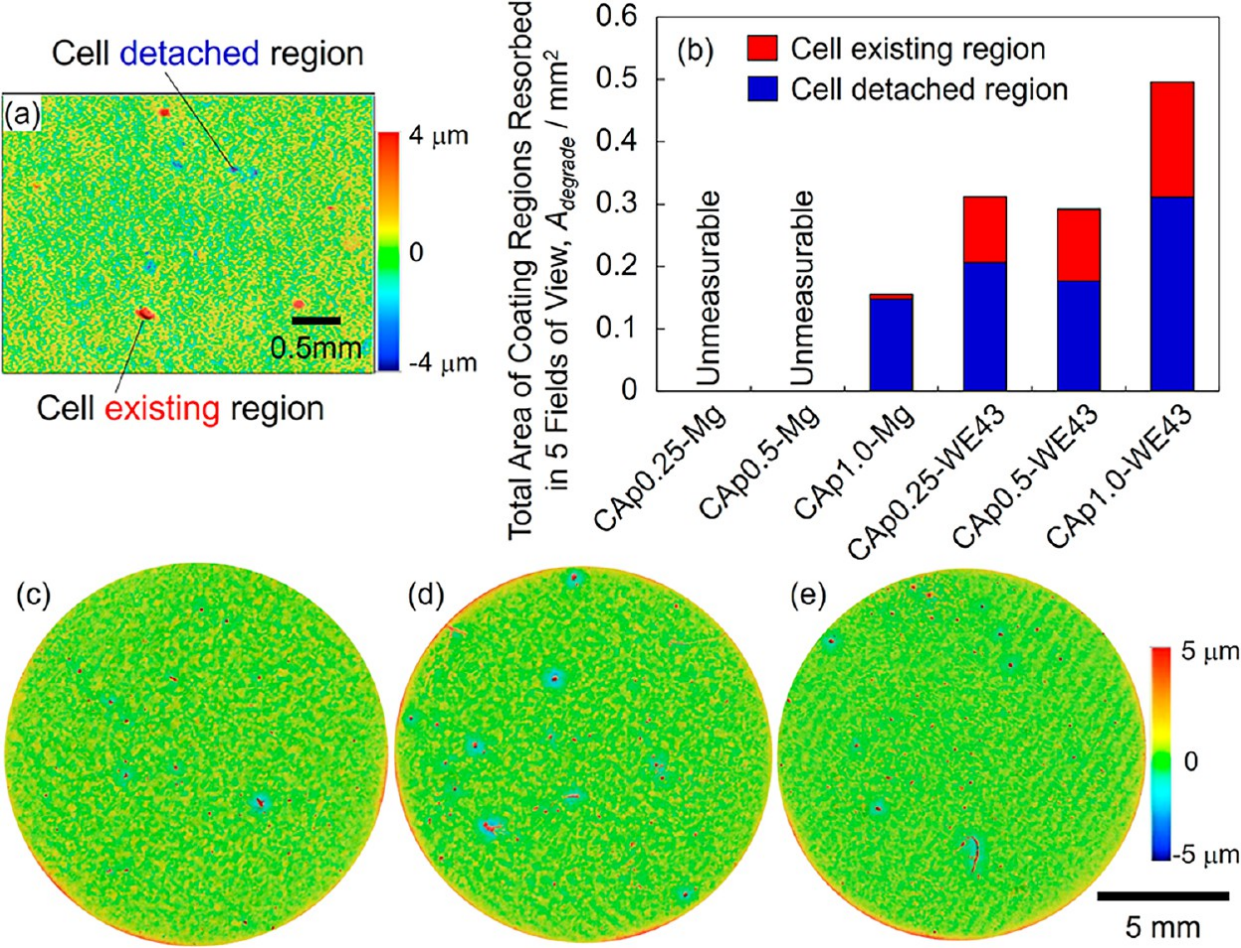
(a) Typical 3D image with osteoclasts on Day 15 of TRAP-stained CAp0.25-WE43. (b) Total area of regions where the CAp coating was resorbed in five fields of view for CAp-Mg and CAp-WE43. 3D images on Day 15 of Giemsa-stained (c) CAp0.25-, (d) CAp0.5-, and (e) CAp1.0-WE43.

Figure 11c–e shows the 3D images of the Giemsa-stained WE43 specimens. In all samples, a cell exhibiting a convex morphology was present at the center of each concave region. Because mature osteoclasts cannot be distinguished from other cells in the Giemsa-stained specimens, the convex cells surrounded by concave areas were considered to be mature osteoclasts, and the concave regions were regarded as areas resorbed by osteoclasts. CAp0.25-, CAp0.5-, and CAp1.0-WE43 exhibited approximately 10, 17, and 15 concave regions, respectively. The size of the concave regions in CAp0.5- and CAp1.0-WE43 was relatively larger than that in CAp0.25-WE43. Furthermore, CAp0.5- and CAp1.0-WE43 exhibited a similar number and size of the concave regions. The low-magnification 3D images of the TRAP-stained WE43 specimens shown in Figure S6 exhibited a similar result to that of the Giemsa-stained samples.

These results demonstrate that osteoclastic resorption of the CAp coating was enhanced by the increased carbonate content. This is attributed to the chemical property of CAp that the higher carbonate content results in higher solubility.^44^ It remains to be further investigated whether the osteoclastic resorbability of the CAp coating on Mg changes with the carbonate content in vivo, as it does in the present in vitro study.

## 4 Conclusions

The corrosion behavior of CAp0.25-, CAp0.5-, and CAp1.0-Mg, containing approximately 11, 17, and 18 wt % carbonate, respectively, was examined by the polarization and EI tests in 0.9% NaCl and Hanks’ solutions. Additionally, the response of osteoclasts to CAp0.25-, CAp0.5-, and CAp1.0-Mg and WE43 samples was evaluated. The findings are summarized as follows:

- In the polarization tests, the *I*_corr_ of CAp-Mg slightly increased with an increase in the carbonate content in a 0.9% NaCl solution. On the contrary, in Hanks’ solution, the *I*_corr_ slightly decreased with increasing carbonate content. The *I*_corr_ values in Hanks’ solution were lower than those in a 0.9% NaCl solution.
- In the EI tests, the CAp coating improved the *R*_p_ of Mg by more than 5000 times in a 0.9% NaCl solution and more than 7 times in Hanks’ solution. The *R*_p_ values in Hanks’ solution were relatively higher than those in a 0.9% NaCl solution.
- The *R*_p_ of CAp-Mg increased by 1.5 times in a 0.9% NaCl solution and 2 times in Hanks’ solution with increasing carbonate content, indicating a reduction in coating defects.
- In culturing osteoclast precursors, CAp1.0-Mg promoted cell proliferation and differentiation into osteoclasts owing to the suppression of substrate Mg corrosion. In contrast, CAp0.25- and CAp0.5-Mg showed significant corrosion, resulting in reduced cell proliferation and differentiation.
- On CAp-WE43, proliferation and differentiation of osteoclast precursors into mature osteoclasts were enhanced with increasing carbonate content.
- The osteoclastic resorption area of the CAp coating increased by approximately 1.5 times with increased carbonate content.

In conclusion, this study demonstrates that the carbonate content of the CAp coating can be used to tailor the corrosion rate of biodegradable Mg alloy devices, such as bone screws, pins, and staples, to accommodate the patient’s age, health condition, and the implantation site. Because degradation of the CAp coating occurs after the induction of osteoclasts or osteoclast-like cells, the coating can maintain its corrosion protection ability until new bone or soft tissue is formed on its surface. Once these cells are induced, CAp coatings with a higher carbonate content are resorbed more rapidly.

## Supplementary Material



## Notes

FTIR spectra for HAp- and CAp-Mg samples, SEM images and EDS surface composition of HAp- and CAp-Mg samples after anodic polarization and EI measurements, high-magnification optical surface images of Giemsa-stained CAp-Mg and CAp-WE43, low-magnification optical surface images of TRAP-stained CAp-Mg and CAp-WE43, and low-magnification 3D images of CAp-WE43 (PDF)

## ⊥

K. M. Nippon Steel Corporation
