# Supplementary material for: Control of Corrosion Resistance and Osteoclastic Resorbability of Bioresorbable Carbonate Apatite Coating for Biodegradable Mg Alloys through Carbonate Content
Source: ACS Biomater Sci Eng. 2025 Dec 1;12(1):322–34. doi: 10.1021/acsbiomaterials.5c01706 (PMC12801190; doi:10.1021/acsbiomaterials.5c01706)
Supplement: Supplementary file 1 [file ab5c01706_si_001.pdf]

Supplemental Material

Control of Corrosion Resistance and Osteoclastic Resorbability of Bioresorbable Carbonate Apatite Coating for Biodegradable Mg Alloys through Carbonate Content

Sachiko Hiromoto [a,b,*], *Kazuma Midorikawa* [a,b,†], *Tomohiko Yamazaki* [c] *and Tomoyuki Yamamoto* [b,d]

[a] Research Center for Structural Materials, National Institute for Materials Science, Tsukuba 305-0047, Japan

[b] Faculty of Science and Engineering, Waseda University, Tokyo, 169-8555, Japan

[c] Research Center for Macromolecules and Biomaterials, National Institute for Materials Science, Tsukuba, 305-0047, Japan

[d] Kagami Memorial Research Institute for Materials Science and Technology, Tokyo 169-0051, Japan

[†] Present Affiliation: Nippon Steel Corporation

* E-mail: HIROMOTO.Sachiko@nims.go.jp

1. FTIR spectra of as-prepared samples

Figure S1 shows the FTIR spectra of HAp-Mg, CAp0.25-Mg, CAp0.5-Mg and CAp1.0-Mg, modified from a figure published in ref. 39. The FTIR spectrum of HAp-Mg exhibits characteristic absorption peaks derived from PO_4^{3-}, H_2O, and OH^- groups in apatite structure along with minor peaks from CO_3^{2-}. The spectra of CAp-Mg show absorption peaks from CO_3^{2-} in addition to those from PO_4^{3-} and H_2O groups.

Because the FTIR measurements were performed using the diffuse reflectance method, the peak intensities do not necessarily correspond to the actual abundance ratios. Nonetheless, the decrease in the phosphate-derived peak intensity around 600 cm^{-1} with increasing carbonate salt concentration of the coating solution suggests an increased substitution of phosphate groups by carbonate groups.

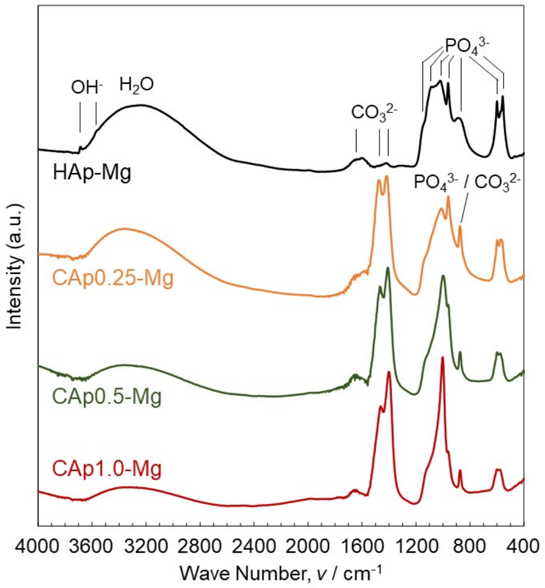

Figure S1. FTIR spectra of as-prepared HAp-Mg, CAp0.25-Mg, CAp0.5- Mg and CAp1.0-Mg samples. Adopted from ref. 39, Fig. 3, with permission.

2. SEM-EDS measurements after polarization and EIS tests

2.2 Surface morphology and composition of samples after anodic polarization

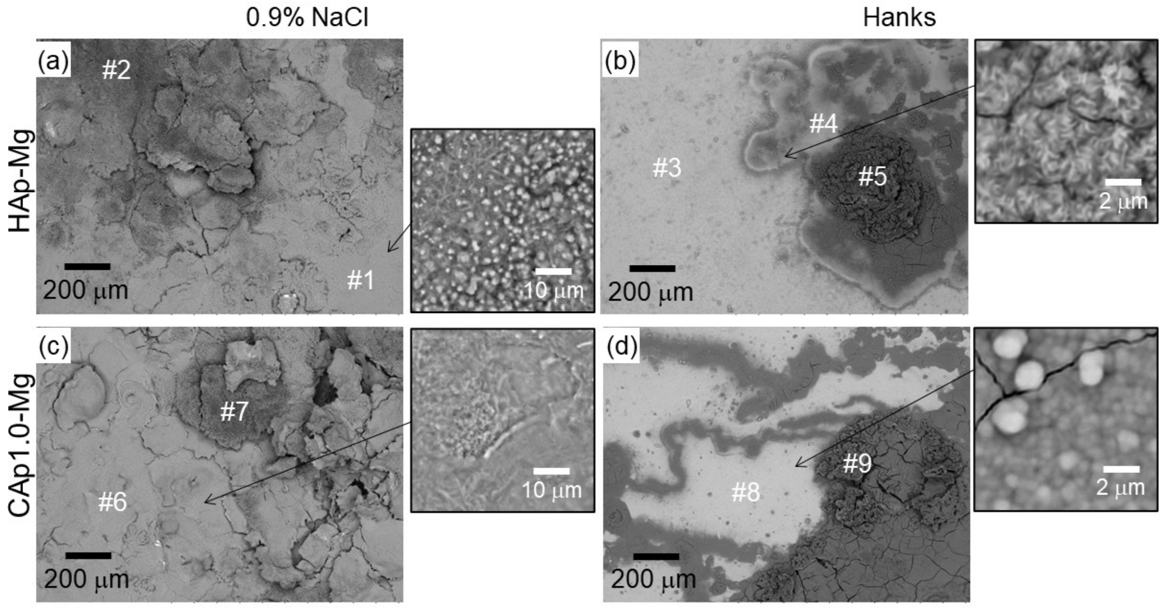

Figure S2. SEM images of (a, b) HAp-Mg and (c, d) CAp1.0-Mg anodically polarized in (a, c) 0.9% NaCl and (b, d) Hanks' solutions. The insets in images show a magnified view of the area indicated by the arrow.

Table S1 Composition of marked areas on the SEM images in Fig. S2.

	(a)		(b)			(c)		(d)	
Sample	HAp-Mg					CAp1.0-Mg			
Test solution	0.9% NaCl		Hanks			0.9% NaCl		Hanks	
Marked area	#1	#2	#3	#4	#5	#6	#7	#8	#9
C	4.91	8.53	7.92	9.46	15.07	5.97	9.58	13.8	18.3
O	64.0	62.0	59.1	61.4	63.8	64.2	61.1	55.9	62.6
Mg	31.0	29.4	3.71	6.96	17.5	30.7	29.0	10.5	16.9
Ca	0.02	-	15.9	12.2	1.83	0.03	0.02	10.4	1.24
P	0.04	-	11.1	8.36	1.37	0.01	0.02	6.53	0.59
Na	-	-	2.08	1.47	0.18	-	-	2.75	0.25
Si	-	-	-	-	-	-	-	0.03	0.01
Cl	-	0.07	0.25	0.18	0.33	-	0.29	0.06	0.08
Ca/P	0.50	-	1.43	1.13	1.34	3.00	1.00	1.59	2.10
O/Mg	2.06	2.11	23. 4	8.82	3.65	2.09	2.11	5.32	3.70

Figure S2 and **Table S1** show the SEM images of HAp- and CAp1.0-Mg and the surface composition for marked areas on the SEM images, respectively, after anodic polarization measurements shown in **Fig. 3**. Due to anodic polarization up to -1.1 V (vs. Ag/AgCl), large corrosion pits were formed on all the samples. In 0.9% NaCl solution, corrosion products covered the surface in apparently not-corroded areas, making the HAp and CAp1.0 coatings invisible (**Fig. S2**(a) and (c)). In Hanks' solution, the original morphology of the HAp and CAp1.0 coatings was observed in not-corroded area (**Fig. S2**(b) and (d)).

On HAp-Mg polarized in 0.9% NaCl, both apparently not-corroded area (#1) and obviously corroded area (#2) were covered with $Mg(OH)_2$ and slight amounts of Ca and P, presumably from the coating, were detected in #1 area. In Hanks' solution, Ca and P were detected in the not-corroded areas (#3 and #4), and small amounts of Ca and P were also detected with $Mg(OH)_2$ even in the significantly corroded area (#5). These results indicate that the corrosion product was $Mg(OH)_2$ in NaCl solution, while in Hanks' solution, in addition to $Mg(OH)_2$, calcium phosphate also precipitated in corrosion sites.

Similar results were obtained on CAp1.0-Mg. The corrosion product of $Mg(OH)_2$ dominantly precipitated on both less-corroded area (#6) and obviously corroded area (#7) in 0.9% NaCl solution, while small amounts of Ca and P precipitated even in significantly corroded area (#9).

2.2 Surface morphology and composition of samples after EI tests

Figure S3 and **Table S2** show the SEM images of HAp- and CAp1.0-Mg and the surface composition, respectively, after electrochemical impedance (EI) measurements shown in **Fig. 4**. No significant changes were observed in the surface morphology before

and after measurements, regardless of the type of coating or test solution. On HAp-Mg, no difference was found in the composition between 0.9% NaCl and Hanks' solutions, while CAp1.0-Mg tested in Hanks' solution showed slightly higher concentration of O, Ca and P than that in 0.9% NaCl solution. This suggests that calcium phosphate can deposit on the surface in Hanks' solution during EI measurements.

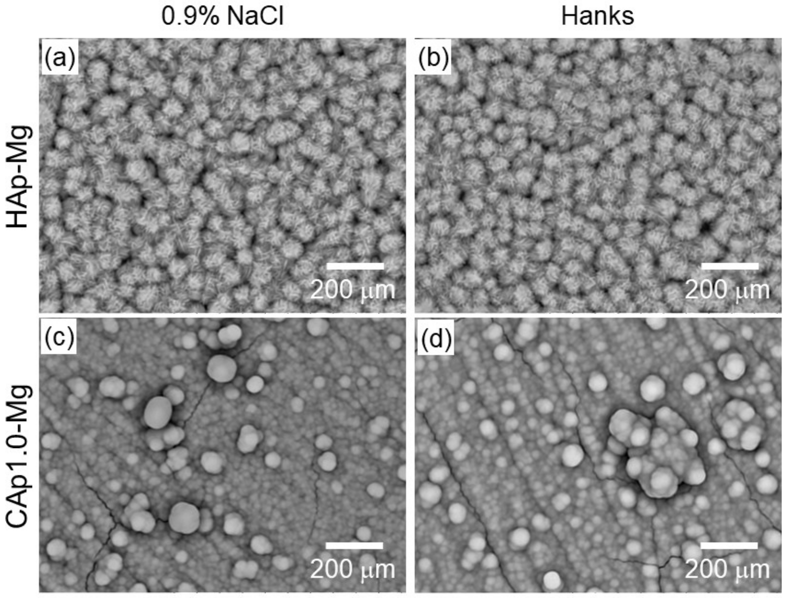

Figure S3. SEM images of (a, b) HAp-Mg and (c, d) CAp1.0-Mg after electrochemical impedance measurements in (a, c) 0.9% NaCl and (b, d) Hanks' solutions.

Table S2 Surface composition of the coatings before and after EI tests.

Sample	HAp-Mg		CAp1.0-Mg	
Test solution	0.9% NaCl	Hanks	0.9% NaCl	Hanks
Composition	(a)	(b)	(c)	(d)
C	6.54 ± 0.14	6.78 ± 0.48	11.3 ± 0.27	10.4 ± 0.28
O	56.5 ± 1.48	59.8 ± 0.97	54.3 ± 4.45	56.9 ± 1.50
Mg	3.89 ± 1.61	2.09 ± 0.81	8.40 ± 4.86	3.62 ± 2.00
Ca	18.1 ± 1.83	16.7 ± 0.46	13.5 ± 0.41	15.5 ± 0.38
P	12.8 ± 0.66	12.0 ± 0.46	8.34 ± 0.15	9.08 ± 0.16
Na	1.98 ± 0.04	2.13 ± 0.06	4.09 ± 0.20	4.36 ± 0.20
Si	0.26 ± 0.29	0.50 ± 0.33	0.18 ± 0.09	0.04 ± 0.02
Ca/P	1.42 ± 0.07	1.39 ± 0.03	1.61 ± 0.81	1.70 ± 0.03

3. Osteoclasts on CAp-Mg and WE43

3.2 Magnified Giemsa-stained images

Figure S4 shows typical Giemsa-stained images of CAp-WE43 and the glass.

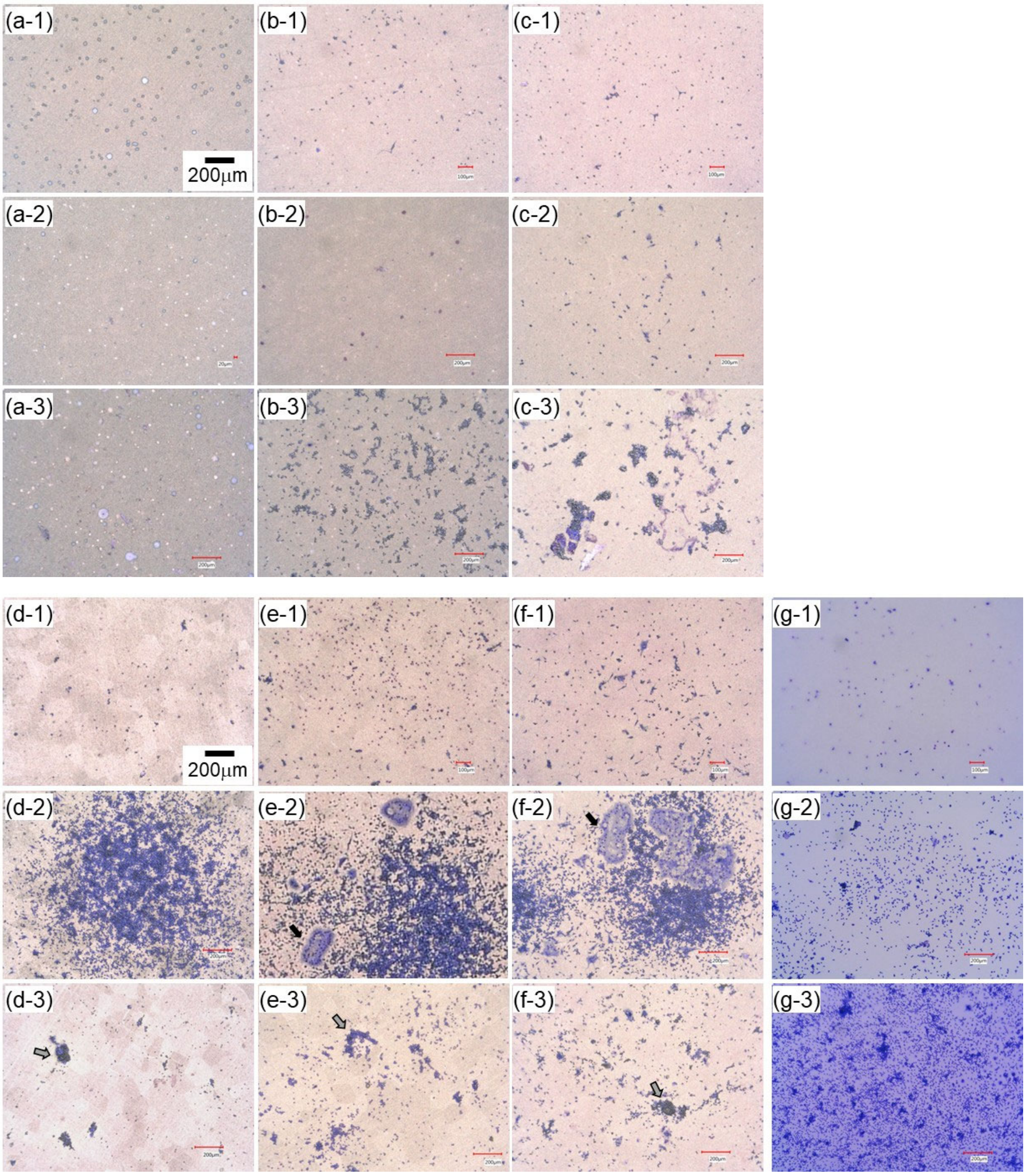

Figure S4. Optical surface images of (a) CAp0.25-Mg, (b) CAp0.5-Mg, (c) CAp1.0-Mg, (d) CAp0.25-WE43, (e) CAp0.5-WE43, (f) CAp1.0-WE43, and (g) glass with Giemsa-stained osteoclast cells. Images on (a-1)–(g-1) Day 4, (a-2)–(g-2) Day 8, (a-3)–(g-3) Day 15. The WE43 images on Day 8 (d-2)–(f-2) were taken by selecting cell colonies. The scale bars are shown in (a-1) and (d-1).

On CAp-WE43, cells were dispersed individually on Day 4, whereas by Day 8, they had formed colonies. Some of the cells have fused to form giant cells, as indicated by arrows in (e-2) and (f-2). This fusion marks both the initiation and progression of maturation into osteoclasts. On Day 8, giant cells were observed on the CAp0.5 and CAp1.0 coatings, but not on the CAp0.25 coating. By Day 15, colonies and giant cells were no longer observed; instead, cell aggregates approximately 100 μm in size, as indicated by arrows, were present. Cell aggregates presumably corresponded to TRAP-positive cells. These observations are consistent with the results obtained from the overall Giemsa-stained and TRAP-stained images.

3.2 Whole surface images of TRAP-stained samples

Figure S5 shows the whole surface optical images of CAp-Mg and WE43 and the glass with TRAP-stained cells on Days 4, 8, and 15. Based on the Giemsa-stained and Calcein-PI images in **Figs. 5** and **6**, it was found that, on CAp-Mg, living cells were rarely observed except in the case of CAp1.0-Mg, due to significant substrate corrosion. Therefore, the discussion will focus on the TRAP-stained images of WE43 and the glass. At the magnification in **Fig. S5**, red TRAP-positive cells were observed on the surfaces of CAp0.5-WE43, CAp1.0-WE43, and the glass on Day 8, and on the surfaces of CAp0.25-WE43, CAp0.5-WE43, CAp1.0-WE43, and the glass on Day 15. In cases of shorter culture durations or lower carbonate content, TRAP-positive cells were not clearly observed. No discolorized regions of the CAp coating were found on Day 8, whereas discolorized areas comparable in size to the cells appeared by Day 15. In contrast, the Giemsa-stained images in **Fig. 5** showed clear colony formation on CAp-WE43 and the glass surfaces at Day 8. On Day 15, the number of cells decreased in CAp-Mg and WE43 compared to Day 8, but cells on the glass remained clearly visible. These results indicate that the osteoclast precursors proliferated between Day 4 and Day 8, but not all cells had differentiated into osteoclasts by Day 8. Furthermore, from Day 8 to Day 15, differentiation and maturation into osteoclasts continued, and the proportion of cells that detached from the surface appeared to increase.

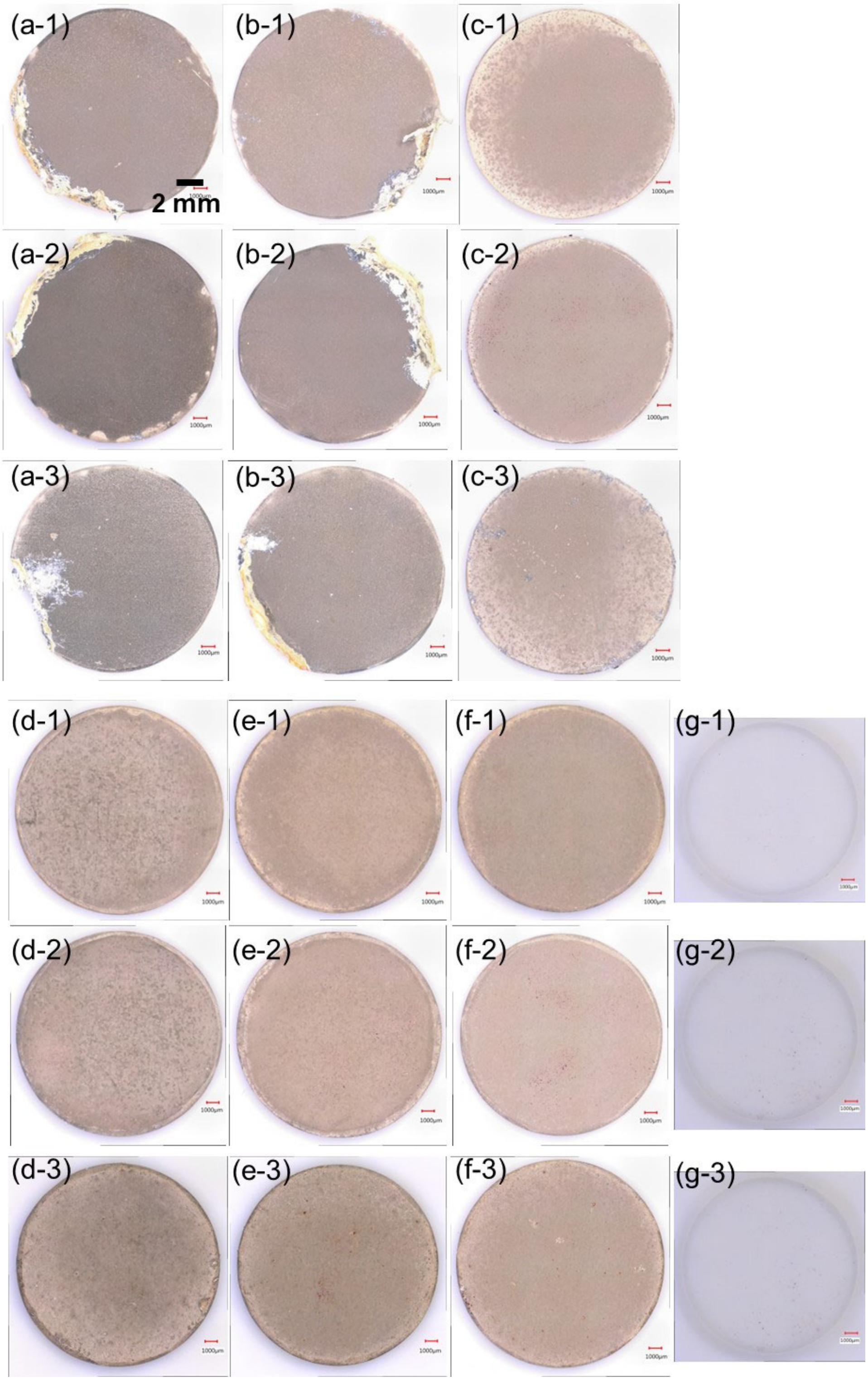

Figure S5. Optical surface images of (a) CAp0.25-Mg, (b) CAp0.5-Mg, (c) CAp1.0-Mg, (d) CAp0.25-WE43, (e) CAp0.5-WE43, (f) CAp1.0-WE43, and (g) glass with TRAP-stained osteoclast cells. Images on (a-1)–(g-1) Day 4, (a-2)–(g-2) Day 8, and (a-3)–(g-3) Day 15. The scale bar is shown in (a-1).

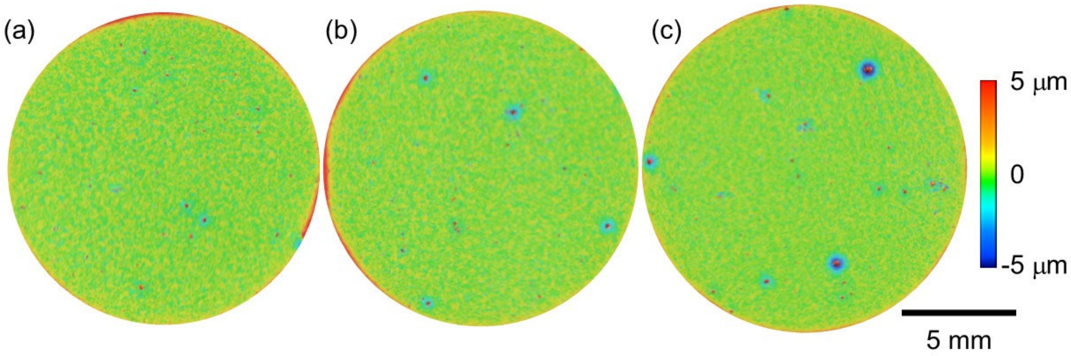

Figure S6. 3D images on Day 15 of (a) CAp0.25-, (b) CAp0.5-, and (c) CAp1.0-WE43 with TRAP-stained osteoclast cells.

Figure S6 shows the 3D images of CAp-WE43 with TRAP-stained cells on Day 15. Because a cell is present at the center of each concave region, these concave regions were considered to be areas resorbed by osteoclasts. The size of the cells in each concave region increased with increasing carbonate content of the CAp coating. Furthermore, the size of concave regions also increased as the carbonate content increased. These findings indicate that increasing the carbonate content leads to enhanced osteoclastic resorption of the CAp coating.
